# No differences in knee kinematics between active and passive flexion-extension movement: an intra-operative kinematic analysis performed during total knee arthroplasty

**DOI:** 10.1186/s40634-020-00229-7

**Published:** 2020-03-12

**Authors:** Alberto Grassi, Nicola Pizza, Nicola Francesco Lopomo, Maurilio Marcacci, Michele Capozzi, Giulio Maria Marcheggiani Muccioli, Francesca Colle, Stefano Zaffagnini

**Affiliations:** 1 II Clinica Ortopedica e Traumatologica, IRCCS, Istituto Ortopedico Rizzoli, Bologna, Italy; 2 Dipartimento Scienze Biomediche e Neuromotorie-DIBINEM, Università di Bologna, Bologna, Italy; 3 Dipartimento di Ingegneria dell’Informazione, Università degli Studi di Brescia, via Branze, 38 25123 Brescia, BS Italy; 4 Humanitas Clinical and Research Center, Milan, Italy; 5 Laboratorio di Biomeccanica, Istituto Ortopedico Rizzoli, via di Barbiano 1/10, I-40136 Bologna, BO Italy

**Keywords:** Total knee Arthroplasty, Total knee replacement, Active and passive flexion, Kinematics, Navigation system

## Abstract

**Purpose:**

The objective of the present study was to acquire and compare by the use of a navigation system the intra-operative flexion-extension movement of the knee performed actively by the patient and passively by the surgeon before and after a total knee arthroplasty (TKA) implantation.

**Methods:**

A cohort of 31 patients with primary knee osteoarthritis (OA), candidate for TKA underwent intra-operative kinematics assessment with a commercial navigation system before and after the definitive implant positioning of a Cruciate Retaining (CR) Mobile Bearing (MB) prostheses. The kinematical data were acquired while surgeon performed the flexion-extension movement (passive ROM - pROM), and while the patient performed it (active ROM - aROM). Differences between pre- and post- implantation and between active and passive motions, were statistically analyzed using paired Student t-tests (*p* = 0.05).

**Results:**

No statistically significant difference were found between aROM and pROM with paired Student t-test regarding internal-external rotation and anterior-posterior translation of the femoral component with respect to the tibia during flexion-extension movement before and after TKA implant (*p* > 0.05).

**Conclusions:**

Active muscle contraction seems to not significantly affect TKA kinematics. The ROM performed by the surgeon during operation resemble the movement actively performed by the patient.

The clinical relevance of this study further supports the use of CAS system in performing intra-operative analysis concerning knee biomechanics.

## Background

The most recent uses of Computer-Assisted-Surgery (CAS) in Total Knee Arthroplasty (TKA) provide the possibility to intra-operatively assess the functional behavior of the knee joint [7–11]. In particular, CAS is able to estimate range of motion and laxities associated with the patient and joint-specific surgical reconstruction. The real-time intra-operative kinematic assessment allows the comparison between the pathological condition (i.e. before the reconstruction) and the newly restored condition (i.e. after the reconstruction). One of the major issues related to the navigation systems concerns its capacity to evaluate only the passive kinematics, hence without taking into account the muscular control of the lower limb. Doro et al. [6], in fact, criticize the lack of active muscular contraction by the patient during the intra-operative evaluation, thus stating that this technology is limited for a proper biomechanical assessment of the knee joint.

Hence, the purpose of the present study was to acquire and compare the intra-operative flexion-extension movement of the knee performed actively by the patient and passively by the surgeon before and after the implantation of a TKA.

The hypothesis was that active knee flexion-extension movement would show comparable pattern to the passive one, demonstrating that even passive kinematics can properly describe the biomechanical behavior of the knee joint.

The clinical relevance of the present study derives from the possibility of evaluating the effects of patient’s active muscular contraction on the kinematics of the osteoarthritic knee and of the TKA hence to better understand the movement of this joint in its complexity.

## Materials and methods

### Patients selection

The study was approved by the ethical committee of the IRCCS Rizzoli Orthopedic Institute (protocol number 11551/CE/US/ml, 5 May 2006).

A cohort of 31 patients with knee osteoarthritis (OA) candidate for TKA was enrolled for the present study after signing an informed consent between 2011 and 2012.

The inclusion criteria were: (1) Primary knee osteoarthritis, (2) Kellgren-Lawrence grade 3–4, (3) BMI < 40 kg/m2. The exclusion criteria were: (1) Previous lower limb alignment corrective surgery on the affected side, (2) BMI > 40 kg/m2, (3) Rheumatoid arthritis, (4) Post-traumatic arthritis.

The mean age of the patients included in the study was 70.5 ± 6.5 years (range 83–54 years), 9 males and 22 females.

### Acquisition protocol

All the patients underwent intra-operative kinematics assessment with a commercial navigation system (BLU-IGS Orthokey, Lewes, Delaware) equipped with a software specifically focused on kinematic analysis (KLEE, Orthokey, Lewes, Delaware) [14]. This system has a 3D RMS volumetric accuracy of 0.350 mm and a 3D RMS volumetric repeatability of 0.200 mm [21], as reported by the producer. All the kinematic data were off-line processed by applying proprietary routines developed in Matlab (Mathworks, Natick, MA, USA).

The proposed methodology was assessed to have a repeatability lower than 2 mm in translation and lower than 3° in rotations [14], with ICC values ranging from 0.94 to 0.99 [4].

Anatomical landmarks on femur and tibia were acquired to define the joint coordinate reference system (JCS) [5, 8] and to perform TKA navigation protocol. The anatomical registration on the femur consisted of: the femoral head (by leg pivoting), the most distal part of the femur in the intercondylar notch (over to the lateral margin of the posterior cruciate ligament), the anterior shaft, the medial and lateral epicondyles, the most posterior and distal part of the condyles and the Whiteside Line (WSL). The medial and lateral malleoli, the tibial spine, the tibial tuberosity and the lateral and medial plateaus were acquired on the tibia.

**Fig. 1:**
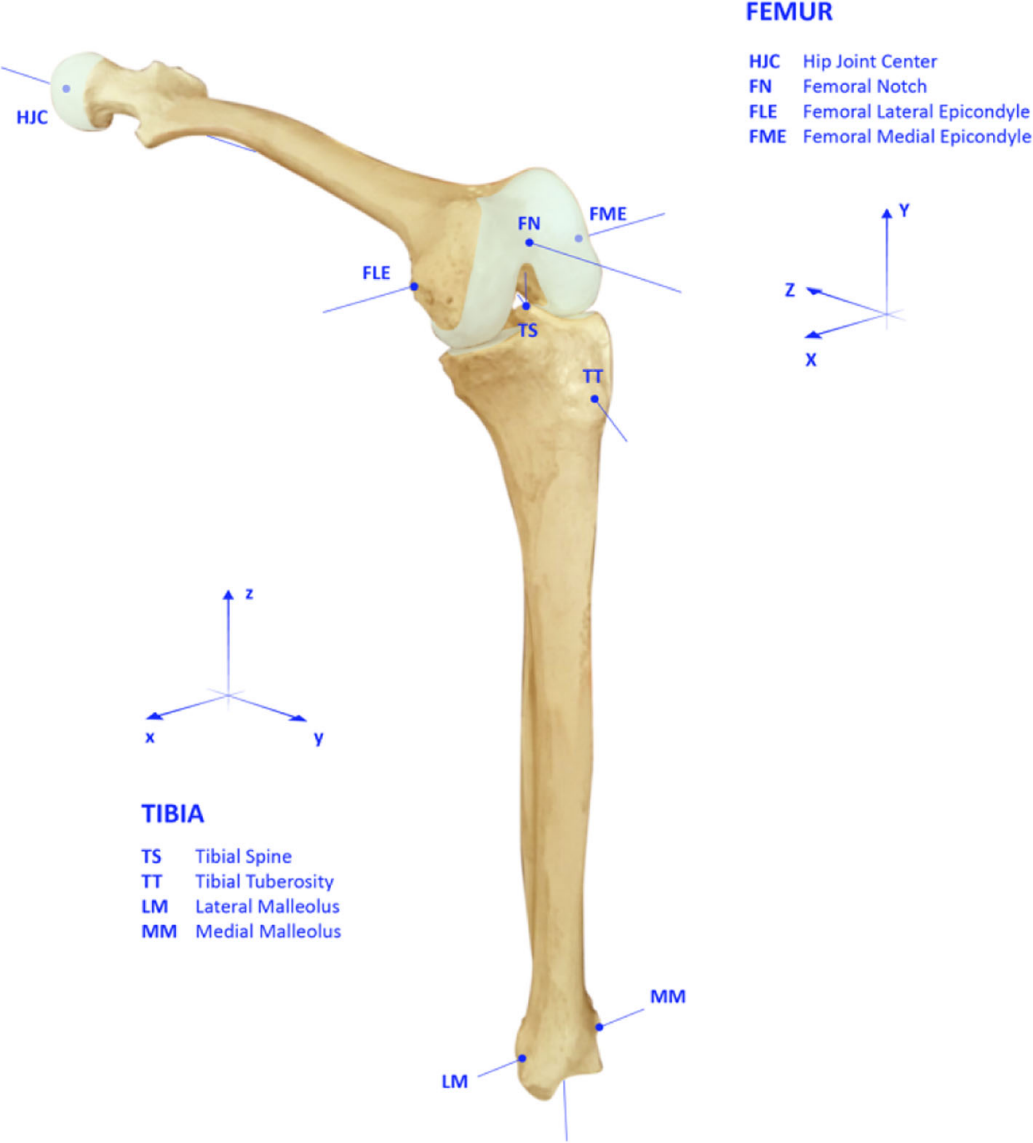
Anatomical reference systems used for the kinematic analysis

Using the anatomical landmarks, the navigation system was able to automatically identify the femoral mechanical axis, surgical trans-epicondylar axis (TEA), WSL, posterior condylar line for femur and tibial mechanical axis and the line connecting medial and lateral tibial plateau for tibia (Fig. 1).

**Fig. 2:**
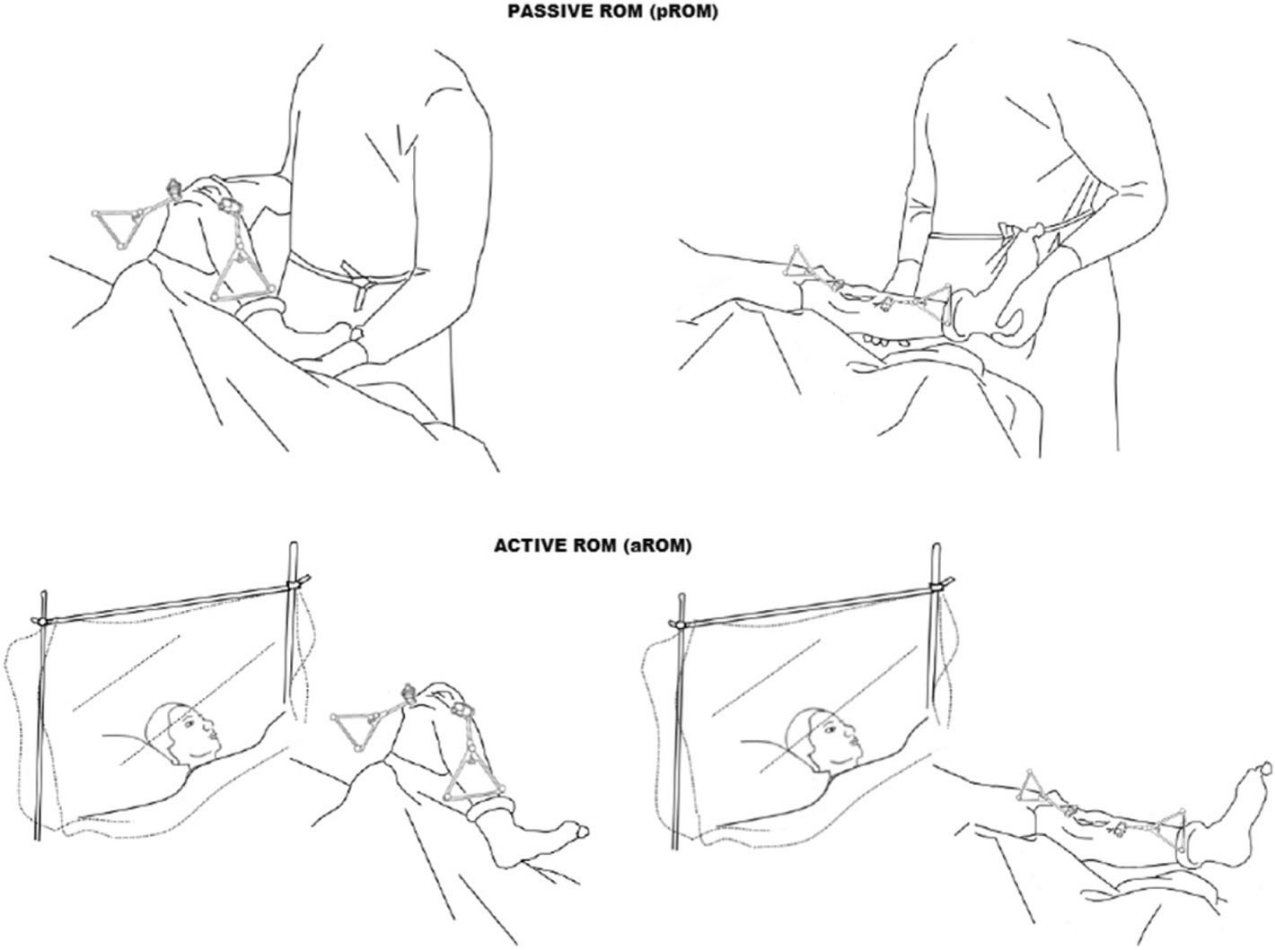
pROM: Passive flexion-extension movement performed by the surgeon (A). aROM: Active flexion-extension movement performed by the patient during surgery in CSE anesthesia

For each patient, intra-operative kinematic acquisitions were collected before and after the definitive implant positioning. Pre-operative kinematic tests were specifically acquired after skin incision to allow the fixation of the tibial and femoral trackers, after medial parapatellar arthrotomy, before patella luxation and meniscal and anterior cruciate ligament (ACL) removal, while post-implant kinematic acquisitions were collected after the cementation of definitive prosthesis. Both the pre-operative and the post-operative acquisition were acquired with the tourniquet inflated, the joint capsule open and patella reduced. The kinematical data were acquired performing flexion-extension movements (full extension-full flexion-full extension), three times for each subject in two different conditions (Fig. 2): the passive motion (pROM), manually performed by the surgeon, maintaining the foot in neutral position (i.e. not introducing any additional stress/torque at foot level during the flexion-extension movement), and the active movement (aROM), directly performed by the patient.

### Surgical technique

All the surgeries were performed under combined spinal and epidural anesthesia (CSE technique) which is a well-known technique typically used during labor. It offers the benefits of rapid onset of analgesia and at the same time allows lower-limb motor power [17]. Therefore, with the use of CSE anesthesia, the patients were able to perform active knee flexion-extension movement after skin incision and tracker positioning for navigation without experiencing pain. A midline skin incision was performed, and both femoral and tibial tracker were positioned in order to not interfere with surgical technique and prevent accidental mobilization. A standard medial parapatellar arthrotomy was performed and the patella was everted. Menisci and ACL were resected, and a tibial cut was made sparing PCL. After the cut of distal femur, the 4-in-1 guide was used to complete the femoral cut with the opportune size. The trial components were positioned and the flexion-extension gaps opportunely balanced, when needed. After the patellar cut and pulsed washing, definitive prothesis was implanted and the tourniquet finally released.

All patients were operated with the standard technique (medial parapatellar approach, adjusted mechanical alignment) and received a cemented Cruciate Retaining (CR) highly congruent Mobile Bearing (MB) TKA (Gemini, Waldemar LINK GmbH & Co. KG, Barkhausenweg 10, 22,339 Hamburg, Germany) with patella resurfacing.

### Data analysis

The coordinate reference system on femur was defined as follows: the femoral mechanical axis as the proximal-distal (PD) axis, the anterior-posterior (AP) axis as the cross product between the PD-axis and the surgical TEA, and the cross product between AP-axis and PD-axis as the medial-lateral (ML) axis, thus achieving an anatomic orthogonal reference system.

The anatomic orthogonal reference system of the tibia was defined as: the PD-axis set as the tibial mechanical axis, the ML-axis as the cross product between the line connecting tibial spine and tibial tuberosity and the PD-axis, and the AP-axis as the cross-product between PD-axis and ML-axis.

Based on the acquired flexion-extension movements, the internal-external (IE) rotations were plotted against knee flexion. The AP translation was computed for both the medial and lateral epicondyles, evaluating their displacement projected in the transverse plane on the tibial reference system.

### Statistical analysis

Starting from the analysis of literature [1, 2], a priori power analysis for a two-tailed paired Student’s t-test (alfa = 0.05, power = 0.8, mean difference of 3.0 ± 5.0° of rotations and 3.0 ± 5.0 mm of displacements) indicated a minimum sample size of 24 subjects.

For statistical comparison of the kinematic behavior, continuous data obtained from passive and active movements from 0° to 120°, both in pre- and post-operative conditions, were re-sampled each 5° of knee flexion using a smooth curve-fitting function that enabled direct comparison of patient.

IE rotations and AP translations values were then averaged on the three repetitions, at every re-sampled angle. The mean values obtained for each subject were then averaged for the whole cohort, thus obtaining one mean curve for the active condition and one for the passive one.

Both in pre- and post-implant conditions, internal-external (IE) rotations and anterior-posterior (AP) translations were estimated for pROM and aROM kinematic tests.

Differences between pre- and post- implantation and between active and passive motions, were statistically analysed using paired Student t-tests (*p* = 0.05). Statistical significance was set at 95% (p = 0.05). Analyse-it software (Analyse-it Software, Ltd., The Tannery 91 Kirkstall Road, Leeds, LS3 1HS, United Kingdom) was used to perform the reported statistical analysis.

## Results

### Tibial IE rotation during flexion

**Fig. 3:**
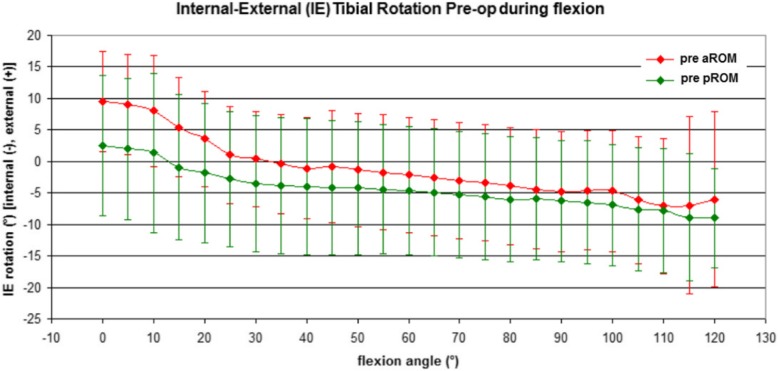
Pre-operative active and passive internal-external rotation of the femur with respect to the tibia

Pre-operative rotation patterns were comparable between active and passive motions, despite aROM showed slightly larger values of internal tibial rotations (Fig. 3).

In fact, in full extension (0° of flexion) aROM showed an average external tibial rotation of 9.5° ± 7.9° while pROM showed an average external tibial rotation of 2.5° ± 11.2°. In early flexion (0°–30°), the screw-home mechanism was observed in both conditions, with an internal tibial rotation of about 9° for active movements and 6° for passive movements. Beyond 30° and up to 120°, tibial rotation showed a further gradual decrease in both aROM and pROM. No significant difference was found between aROM and pROM with paired Student t-test (n.s.).

**Fig. 4:**
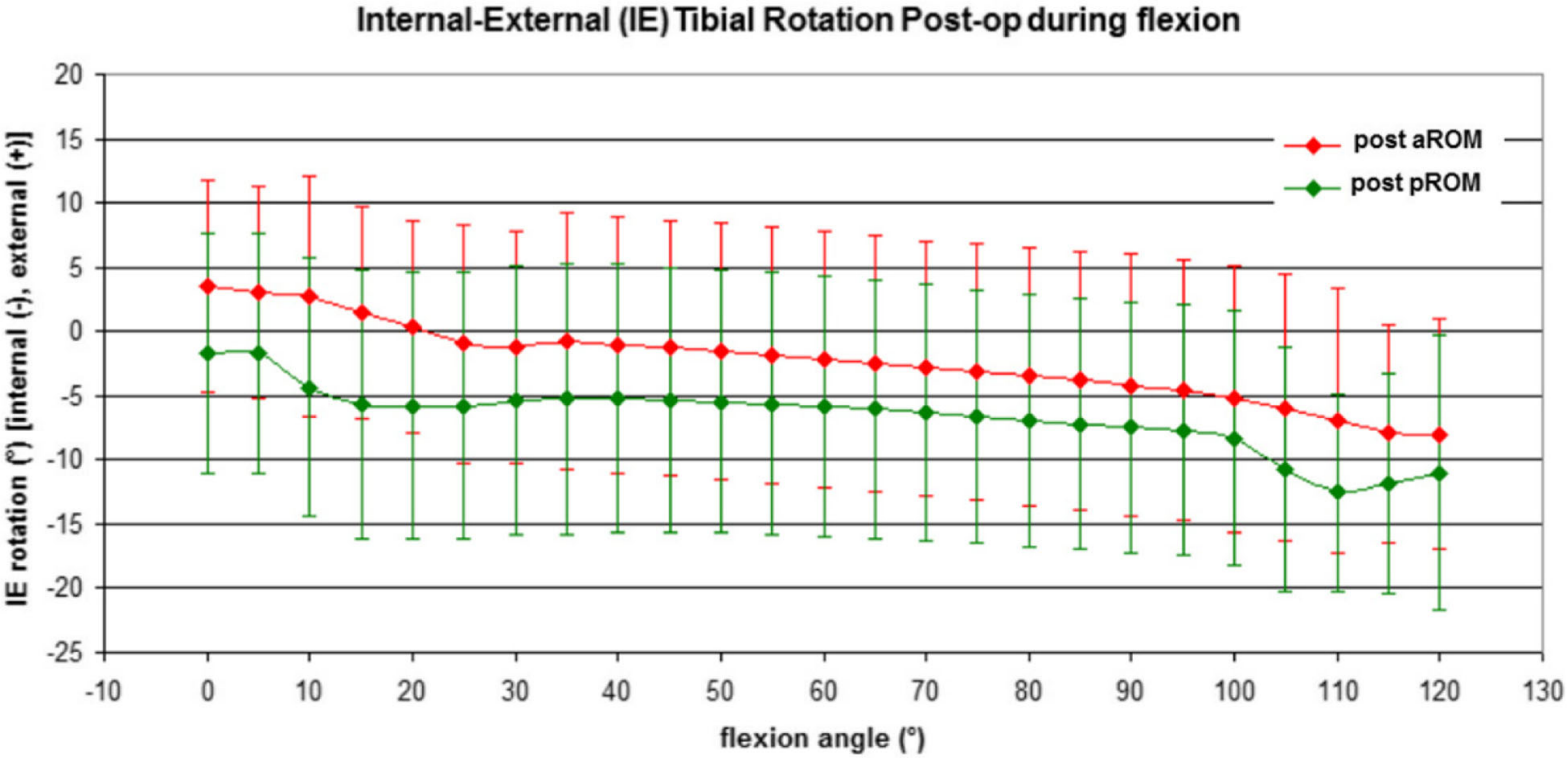
Post-operative active and passive internal-external rotation of the femur with respect to the tibia

Also, the post-operative rotation patterns were similar for active and passive motions (n.s.), showing a screw-home mechanism with an internal rotation of about 4° for both curves (Fig. 4).

The post-operative IE rotation was reduced compared to pre-operative status for both aROM and pROM, however without statistical significance (n.s.).

### Femoral AP translation during flexion

**Fig. 5:**
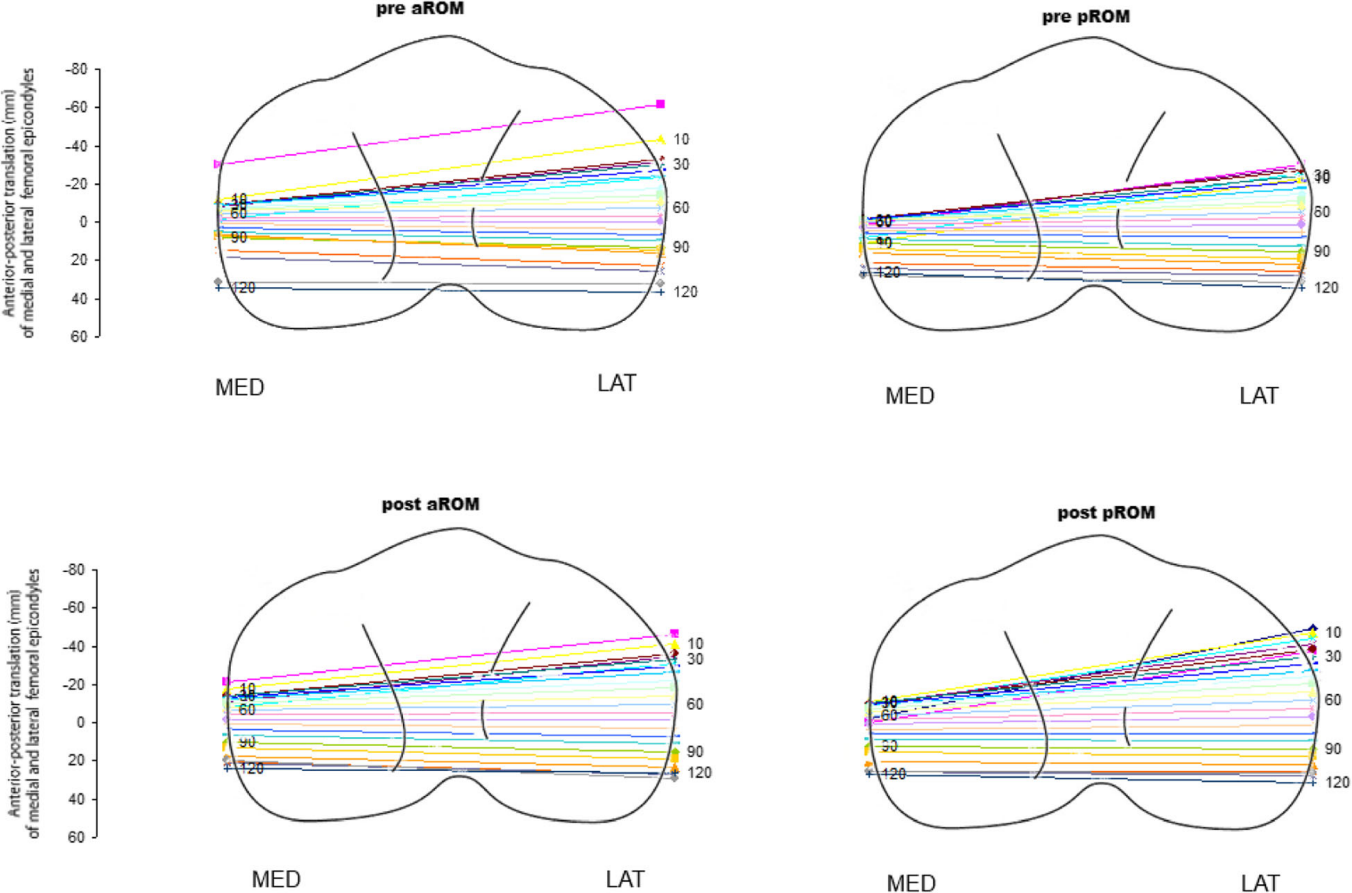
Pre-operative (top) and post-operative (bottom) active and passive antero-posterior translation of the femur with respect to the tibia

Pre-operative translation of the medial and lateral femoral condyles presented similar pattern in all conditions under study (Fig. 5, top).

The greater anterior translation was registered for lateral compartment, with a mean value of 63.5 ± 20.8 mm for aROM and 52.2 ± 26.9 mm for pROM. Differently, the medial compartment had a smaller mean displacement of 33.8 ± 37.3 mm for aROM and 18.6 ± 29.4 mm for pROM. For all patients, the translation occurred between 0° and 90° of flexion.

In the post-operative status (Fig. 5, bottom) after implant positioning, a slightly increased anterior displacement of the femur was found for lateral compartment during aROM (72.6 ± 29.4 mm) and pROM (71.9 ± 26.9 mm) with respect the pre-operative values. The displacement of medial compartment was 35.4 ± 48.3 mm for aROM and 32.4 ± 30.2 mm for pROM. No differences were registered between the pre-operative and post-operative status for both aROM and pROM (*p* > 0.05).

## Discussion

The most important finding of the present study was that there are no significant differences between a flexion-extension movement actively performed by the patient and the same movement passively performed by the surgeon before and after TKA implantation.

The use of a navigation system in TKA has been proven to be extremely useful to perform a reliable intra-operative assessment of joint kinematics [7, 9–12, 15, 18, 19]. However, so far, the presence or not of the muscle contraction still remained un-investigated, ignoring if it could affect intra-operative joint kinematics [6].

The main finding of the present study suggest that the passive ROM performed by the surgeon allows a reliable assessment of the knee kinematics; further, in this specific setup, muscle contraction do not significantly affect knee kinematics. Analyzing the available literature there was no study conducted in such scenario, and just few papers, to authors knowledge, compared post-operative aROM with post-operative pROM. Laidlaw et al. highlighted that the active ROM was significantly lower than passive ROM in a cohort of patients with a CR TKA design (aROM 100.3 degrees; pROM 115.2 degrees; *p* < 0.001). The aROM was radiographically assessed at maximum flexion meanwhile the pROM clinically with a goniometer [13]. Song et al. compared passive maximum flexion without weight-bearing and other four flexion types, included active non-weight-bearing. They concluded that a greater maximum grade of knee flexion after TKA was achievable with the passive ROM with respect to the active ROM. However, this study was just focused on measuring the maximum degrees of flexion without any mention to the kinematical behavior during every task [20]. Although those results seem to be in contrast with the ones of the present study, it must be taken into account that substantially different methods were used, hence it is difficult to directly compare results and draw any solid conclusion.

Another notable finding of the present study was that TKA design restored the physiological screw home mechanism in early flexion (0°–30°). The tibia performed an internal rotation of about 9° during the active movement and 6° during the passive one in pre-operative assessment, meanwhile after TKA implantation of 4°. Nevertheless, the comparison of pre and post-operative tasks seems to be in contrast with another kinematical study. Mooroka et al. [16] infact, analyzed pre and post-operative knee kinematic with CAS, finding out that before and after a Posterior Stabilized TKA implantation the physiological knee motion was not present.

An important aspect to consider is that the data showed in the present study had been acquired without weight bearing on the analyzed limb. Recently, instead, it has been demonstrated that significant differences in TKA kinematics occur during weight bearing respect to non-weight-bearing conditions. Bragonzoni et al., in fact, observed that in weight-bearing condition, the prosthesis femoral component had a significantly wider internal rotation during chair-raise instead of during an active ROM [3]. Therefore, it seems that more than muscle contraction is the weight bearing that affects the knee kinematic during a flexion-extension movement. This could be an explanation why in the present study no significant differences were found between pROM and aROM despite patient’s muscle contraction.

The clinical relevance of the present study is that the pROM performed by the surgeon during surgery can accurately resemble the aROM with muscle contraction by the patient in open kinematic chain. This result comes to further support the use of CAS system in performing intra-operative analysis concerning knee biomechanics, thus, to provide fundamental information on both joint conditions and surgery procedures.

The findings of this study have to be seen in the light of some limitations. Both aROM and pROM data were acquired after joint capsule were opened, so it could have possibly altered the kinematical behavior of the knee, due to the loss of its role of joint restrainer. However, this condition was present in both evaluations and therefore it is possible that this factor did not affected too much the primary endpoint. The presence of the tourniquet could have affected the kinematical evaluation in particular the aROM acquisition. The post-operative data presented refer to a specific prothesis design, hence other TKA designs could give different results. The intra-operative assessment of the ROM still remains an empirical evaluation of the knee movement which, for sure, resemble the daily living movement but do not completely investigate the knee motion in all his complexity. Finally, despite the power analysis indicated a minimum sample size of 24 subjects with a larger cohort the differences detected could become statistically significant even though the clinical significance has to be defined.

Despite these limitations, to the best knowledge of the authors this represents the first study comparing active ROM performed by the patients intra operatively and the same movement performed by the surgeon and poses the basis for further studies that compares intra-operative kinematic with active daily life motor tasks in order to increase the knowledge on knee biomechanics.

## Conclusion

No significant differences between a flexion-extension movement actively performed by the patient and the same movement passively performed by the surgeon before and after TKA implantation were detected during an intra-operative kinematic evaluation using a navigation system. This result comes to further support the accuracy of the CAS system in intra-operative knee kinematic evaluations without the need for patient active contraction which seems to not affect the knee kinematics in the described setting.

## Data Availability

All data generated or analysed during this study are included in this published article.

## Abbreviations

ACL: Anterior Cruciate Ligament
AP: Anterior/Posterior
aROM: Active Range of Motion
CAS: Computer-Assisted-Surgery
CR: Cruciate Retaining
CSE: Combined Spinal and Epidural
IE: Internal/External
JCS: joint coordinate reference system
MB: Mobile Bearing
ML: Medial/Lateral
OA: osteoarthritis
PD: Proximal/Distal
pROM: Passive Range of Motion
TEA: trans-epicondylar axis
TKA: Total Knee Arthroplasty
WSL: Whiteside Line
